# The risk of fatal bleeding complications in jugular catheterization in patients with coagulopathy: A retrospective analysis of death cases in closed claims and the Medical Accident Investigating System in Japan

**DOI:** 10.1371/journal.pone.0261636

**Published:** 2022-01-13

**Authors:** Yasuhiro Otaki, Naofumi Fujishiro, Yasuaki Oyama, Naoko Hata, Daisuke Kato, Shoji Kawachi

**Affiliations:** 1 General Medical Education and Research Center, Teikyo University, Tokyo, Japan; 2 Safety Control Department, Teikyo University Hospital, Tokyo, Japan; 3 Specialty Claims Department, Sompo Japan Insurance Incorporated, Tokyo, Japan; Medical University Innsbruck, AUSTRIA

## Abstract

**Background:**

To prevent recurrence of medical accidents, the Medical Accident Investigating System was implemented in October 2015 by the Japan Medical Safety Research Organization (Medsafe Japan) to target deaths from medical care that were unforeseen by the administrator. Medsafe Japan analyzed the 10 cases of central venous catheterization-related deaths reported in the system and published recommendations in March 2017. However, the particular emphasis for the prevention of central venous catheterization-related deaths is unclear.

**Methods:**

This study aimed to identify the recommendation points that should be emphasized to prevent recurrence of central venous catheterization-related deaths. We assessed central venous catheterization in 8530 closed-claim cases between January 2002 and December 2016 covered by the medical insurer Sompo-Japan. Moreover, we compared central venous catheterization-related death in closed-claim cases with death in reported cases.

**Results:**

The background, error type, anatomic insertion site, and fatal complication data were evaluated for 37 closed-claim cases, of which 12 (32.4%) were death cases. Of the 12 closed-claim cases and 10 reported cases, 9 (75.0%) closed-claim cases and 9 (90.0%) reported cases were related to vascular access. Among these, 5 closed-claim cases (41.7%) and 7 reported cases (77.8%) were related to internal jugular vein catheterization (p = 0.28). Coagulopathy was observed in 3 (60.0%) of 5 closed-claim cases and 6 (85.7%) of 7 reported cases.

**Conclusions:**

The risk of internal jugular catheterization in patients with coagulopathy must be carefully considered.

## Introduction

The Medical Accident Investigation System was implemented by the Japan Medical Safety Research Organization (Medsafe Japan) in Japan in October 2015 [1]. This system targets unforeseen death caused by medical care that was reported as a *medical accident*, defined as “Death or stillbirth which are caused or suspected to have been caused by the care provided by employees of the medical institutions, and which are unforeseen by the administrator” [2]. This system aims to ensure medical safety by preventing the recurrence of similar medical accidents. In this system, each medical institution prepares and submits an In-Hospital Investigation Report to the Medical Accident Investigation and Support Center (ISC) of Medsafe Japan. The ISC then compiles and analyzes these reports to develop recommendations for preventing the recurrence of similar medical accidents. In March 2017, Medsafe Japan and the ISC released the recommendations with the “Analysis of deaths related to the complications of ‘Central Venous Catheterization’: First report” published as the first set of recommendations due to the repeated occurrence of unforeseen deaths associated with central venous catheterization (CVC)-related complications [3]. In this publication, 10 in-hospital investigation reports of the deaths were carefully analyzed in detail, and the results of the analysis were combined with the conventional findings. The recommendations include 9 items for preventing accidents, of which 6 were related to the indications for and practice of CVC, such as anatomic insertion site focusing on the internal jugular vein, puncture procedure using the ultrasound-guided method, and recommendations for avoiding fatal complications. In October 2017, 6 months after the publication, the CVC recommendations were used in manual reviews and training materials in many hospitals in a questionnaire-based survey, which was conducted by Medsafe Japan [4]. Although the recommendations are important to prevent the recurrence of similar CVC-related medical accidents, which specific recommendations to emphasize for prevention is unclear because the 10 deaths have not been compared with other CVC-related medical cases.

For physicians, a reduction in CVC complications is always a priority. Although CVC is an invasive medical procedure that is widely performed in children and adults, in numerous cases, incorrect indications or insertions using premature techniques can compromise patient safety with disastrous consequences. To properly perform CVC and achieve its original objectives, several clinical guidelines have been established [5–7], including the CVC guidelines of the Japan Society of Anesthesiologist [8]. Furthermore, to reduce the incidence of CVC complications, studies have focused on specific complications [9–11], including the study by Parienti *et al*. on the incidence of catheter infections and occlusions [12]. In addition, Ares *et al*. reported the indications, devices, and risks of CVCs in children [13], and Norris *et al*. [14] and Jaffray *et al*. [15] reported the complications of CVCs in children. Domino *et al*. analyzed the closed claims of CVC-related complications and identified various potentially fatal complications [16]. Closed claims held by insurers are currently used among different medical fields, including anesthesiology, to improve medical safety by preventing medical accidents [17–19]. We have also analyzed closed claims provided by Sompo-Japan (SJ), a leading insurer that covers >70% of all medical facilities in Japan, including various types of hospitals and clinics. These data report the characteristics of medical accidents in rheumatoid arthritis and suppurative arthritis to improve patient safety [20, 21].

Although closed claims are thought to represent only a portion of medical accidents and have a bias toward medical accidents that resulted in serious injuries, closed claims contain data on different types of medical accidents ranging from non-serious injuries to deaths as well as rare fatal medical accidents [22]. Therefore, these data may elucidate the recommendation points that should be emphasized to specifically prevent the recurrence of similar CVC-related deaths, including unforeseen death, by comparing CVC-related closed-claim cases (CCs) with CVC-related reported cases (RCs) documented as unforeseen deaths in the Medical Accident Investigation System. In this study, we aimed to compare and analyze CCs and RCs to identify points in the recommendations published by Medsafe Japan and ISC that should be emphasized specifically to prevent the recurrence of similar CVC-related deaths.

## Materials and methods

### Study design

This retrospective comparative study was conducted to review CVC-related closed malpractice claim cases treated at the Tokyo headquarters of SJ, defined as the *closed-claim cases* (*CCs*), and CVC-related death cases reported in the Medical Accident Investigation System, defined as the *reported cases* (*RCs*). This study complied with the Japanese epidemiologic study guidelines and was approved by the ethics committee of our university; Teikyo University Ethical Review Board for Medical and Health Research Involving Human Subjects (authorized number: Teirin19-059). To preserve anonymity in the present study, all claim files underwent a contextual de-identification process by SJ staffers before being provided to the reviewers. Therefore, the ethics committee waived the requirement for informed consent in this retrospective study.

### Closed-claim cases from Sompo-Japan

The present study evaluated CCs related to CVC-related medical practices that were processed by professional staff at SJ over 15 years, between January 2002 and December 2016. The CCs analyzed in this study included data on various medical accidents, ranging from non-serious injuries to deaths. The coverage by SJ extends to >70% of all medical facilities in Japan, including various types of hospitals and clinics. This study was conducted at the Tokyo headquarters of SJ, which handles the highest number of claims within the company as a centralized library of claims for all of Japan. A *claim* was defined as a written statement demanding compensation for injuries caused by medical practice. [23] Claims were classified as *closed* if they were dropped, dismissed, or settled by monetary compensation after reconciliation or a judicial decision [24]. Claim files provided by the insurer contained various types of relevant information, including the initial reports from the insured party when the allegations arose; legal reports, such as judgment documents; expert opinions; and relevant medical records obtained from medical facilities [21]. A total of 8530 closed claims were processed at the Tokyo headquarters during the 15-year study period. Of these 8530 claims, 37 CVC-related claims (0.4%) were retrieved for this study. All the cases treated at the Tokyo headquarters of SJ during the period were included in the analysis, regardless of the judgment results. There was no overlap between CCs from SJ and the RCs from the Medical Accident Investigation System.

### CVC-related death cases from the Medical Accident Investigation System

In October 2015, Medsafe Japan initiated the Medical Accident Investigation System to prevent the recurrence of similar medical accidents in Japan. In this system, unforeseen deaths caused by medical care were reported as medical accidents. In this system, *medical accident* is defined as “Death or stillbirth which are caused or suspected to have been caused by the care provided by employees of the medical institutions, and which are unforeseen by the administrator” [2]. If the case is judged to be a medical accident, the administrator explains the judgment to the bereaved family and reports it to the ISC. From October 2015 to December 2016, 226 cases were reported to the ISC as unforeseen death. Of these 226 cases, 10 (0.4%) were CVC-related death cases. Subsequently, in March 2017, Medsafe Japan and the ISC published “Analysis of deaths related to the complications of ‘Central Venous Catheterization’: First report” as the first set of recommendations for the prevention of recurrence of similar medical accidents. These recommendations include nine items to prevent CVC-related accidents (Table 1). In this study, we analyzed the characteristics of the reported death cases described in the recommendations.

**Table 1:** Recommendations for the prevention of recurrence of medical accidents (number 1)–analysis of deaths related to the complications of “central venous catheterization”: First report*.

Number	Recommendation
1	It is essentially important to become aware that Central Venous Catheterization (CVC) is a hazardous medical intervention having a possibility of fatal complications. Especially, a patient with blood coagulation disorder or with intravascular dehydration, has a high potential danger of death and the CVC intervention should be decided after careful discussion, keeping in mind a possibility of substitution of Peripherally Inserted Central Catheter (PICC).
2	Prior to the catheterization, the patient should be explained its necessity and give consent to the specific risk peculiar to him-/herself as well, and that should be recorded in writing. Especially in the case of serious illness, if CVC is indispensable even after considering the risk of death, it is important for the physician to explain the risk sufficiently and to obtain understandings from the patient or family.
3	At the start of intervention to the internal jugular vein, it is recommended to perform ultrasound “Pre-Scan”, for identifying the vein and its appearance (its diameter, collapsed or not), its position (the depth from the skin), and the anatomical relationship to the artery.
4	“Real-time ultrasound-guide” has become an essential assisting method for CVC, but at the same time, it has a “Pitfall” that could misguide toward serious complications. It is advised that the operator should receive a training on the simulator in advance.
5	The needle in the “CVC kit” is mostly too long for the internal jugular vein. Therefore, do not insert beyond the reach of jugular vein. Especially in the case of emaciated patient, the operator should pay attention not to insert too deep.
6	During the intervention, confirm that the guide wire is in the lumen of intended vein by ultrasound or X-ray fluoroscopy. Especially in the route of internal jugular vein, the inserted guide wire should not exceed 20 cm in order to reduce the occurrence of arrhythmia and vein wall injury by the guide wire contact.
7	If sufficient reverse aspiration from the indwelling catheter cannot be seen, the catheter should not be applied as a general rule. Particularly in the case of intravenous double-lumen catheter for dialysis, it is mandatory to confirm the position of the catheter because the malposition of the catheter may cause fatal complications.
8	In the management after the catheter insertion into the central vein, careful observation is necessary, keeping in mind the possibility of fatal complications. If the patient shows newly developed signs, such as a decrease in blood pressure, dyspnea, restlessness, and an unnatural reverse flow in the infusion line, it is necessary to promptly examine and diagnose the possibility of hemothorax, pneumothorax, and airway narrowing as well as of the catheter tip malposition. Physicians and nurses should share all the information and observe the patients’ condition, including problems at the time of intervention.
9	In order to respond promptly to the event of complications, the cooperation with other departments including transfer to other hospitals should be designed in manual.

*Six items in the recommendations were related to the indication (no. 1) and practice for CVC, such as anatomic insertion site focusing on the internal jugular vein, puncture procedure under ultrasound guidance, and recommendations for avoiding fatal complications (nos. 3–7).

### Outcome measures

The background for CCs and RCs were compared. First, the descriptive statistical analysis considered the demographic data, such as disease for CVC, reason for CVC, and insertion site, for both CCs and RCs. The ratio of ultrasound guidance in CVC and the involvement of anesthesiologists in CCs were also analyzed. Second, the error type was analyzed for both CCs and RCs. The errors were divided into *vascular access errors* and *use/maintenance errors*, and the ratio of death cases in each error type was analyzed. Third, the anatomic insertion site and fatal complication types in the death cases were analyzed for both CCs and RCs. For the analyses of the anatomic insertion site and complication type, the insertion sites were classified as *jugular*, *subclavian*, and *femoral*, and the relationship between these sites and the fatal complication types was assessed. The judgments of each outcome were determined independently by the two co-authors of this study to control for potential bias from the reviewers’ personal interpretations.

### Statistical analyses

Descriptive statistics were used to evaluate the data. Statistical significance, defined as a p value of <0.05, was determined using the Fisher exact test. Statistical analyses were conducted using R version 4.0.3 (R Core Team [2020], R: A language and environment for statistical computing, R Foundation for Statistical Computing, Vienna, Austria, http://www.R-project.org/).

## Results

### Background of the closed-claim cases and reported cases

In this study, we analyzed 37 CCs and 10 RCs. Summaries of RCs from the recommendations are presented in Table 2. The baseline data of CCs and RCs are presented in Table 3.

**Table 2:** Summaries of reported cases from recommendations.

Case number	Case summary
Case 1	The patient suffered from disseminated intravascular coagulation syndrome, occurred during advanced cancer chemotherapy. Difficulty in communication. The cause of death was suffocation due to cervical hematoma. Ai data: present, autopsy data: absent. For the purpose of infusion therapy to improve the general condition, insertion of the central venous catheter through the right internal jugular vein was tried with the aid of the ultrasound-guided pre-scan, but the carotid artery was punctured and treated with astriction. Subsequently, CVC was attempted in the left internal jugular vein with the real-time ultrasound-guided method, but the catheter did not move ahead. After removal, hematoma was detected and treated with astriction again. Respiratory stenotic sounds were heard at 10 minutes after the end of the procedure, and in another 50 minutes, the rightward deviation of the trachea was confirmed by chest X-ray. Immediately after that, decreased breath sounds and immeasurable low blood pressure were observed, and the patient died.
Case 2	The patient was at the terminal cirrhosis, with bleeding tendency. The cause of death was the bleeding in upper mediastinum and right side haemothorax, due to right vertebral artery injury. Ai data: present, autopsy data: present. In order to correct hypokalaemia, CVC (triple lumen catheter) to the right internal jugular vein was done with the aid of the ultrasound-guided method. But when the guide wire was inserted, there was a resistance against the inserted guide wire, so it was once removed. Although there was a complaint of dyspnea during the re-intervention, the catheter was inserted smoothly with no ultrasound findings suspected of pneumothorax. From 15 minutes after the end of the procedure, decrease in SpO2 and blood pressure were observed. With the treatment of infusion and blood transfusion implemented, there was no improvement. And the intensive treatment with respirator, continuous hemofiltration, etc. were followed, but the patient died three days after the CVC.
Case 3	Because of the advanced age, the patient had difficulties in eating and also in communicating. The cause of death was a change in hemodynamic related to pneumothorax and suspected haemothorax. Ai data: absent, anatomy data: absent. Because of the difficulty in securing the peripheral blood vessels for infusion, the interventions of CVC were done multiple times to the internal jugular vein and the subclavian vein by the landmark method. The intervention did not succeed in securing the insertion. Several arterial punctures were observed. Another insertion was tried from the inguinal region but failed and discontinued. The chest CT taken approximately 40 minutes after the procedure showed right pneumothorax, and the aspiration had no effect, resulting in cardiopulmonary arrest and death.
Case 4	The patient was in the condition of post-hepatectomy and under treatment with heparin for the portal vein thrombosis. An emergency surgery was performed for the panperitonitis due to perforation of duodenum. The cause of death was suspected of right side haemothorax immediately after the removal of the catheter. Ai data: absent, autopsy data: absent. For managing the general condition, CVC (double lumen catheter) was performed to the right internal jugular vein with the aid of the ultrasound-guided method in the operating room under general anaesthesia. It was confirmed that the position of the catheter tip was judged in good position and no problem on chest X-ray. The infusion started. Next morning, because of the decreased permeability in the right lung on the chest X-ray and decreased SpO2, the infusion was discontinued, and thoracic cavity drainage was performed. CT showed that the catheter tip was deviated into the thoracic cavity, but it could not be judged the catheter was running through the artery. After removal of the catheter, the patient fell into shock in several minutes and underwent emergency thoracotomy, but later died.
Case 5	The patient had suffered from ulcerative colitis. The cause of death was lethal arrhythmia induced by the diastolic disturbance of heart due to cardiac tamponade. Ai data: present, autopsy data: present. For the purpose of intravenous hyper-alimentation, a femoral catheter (60 cm length) was inserted into the right subclavian vein with the assist of Landmark method and fixed at 25 cm. Because the chest X-ray showed no problem, intravenous hyper-alimentation was started. Abnormal backflow was observed occasionally through the infusion line. After two weeks, the patient showed dysphoria and fell into shock. On the CT, the catheter tip was located in the right ventricle and at the same time, cardiac tamponade was detected. When the catheter was withdrawn 5 cm, ventricular fibrillation occurred immediately after. The patient was transferred to another hospital with continuing cardiopulmonary resuscitation but died on the same day.
Case 6	The patient had been under the treatment of maintenance hemodialysis for chronic renal failure and taking anticoagulant drugs for atrial fibrillation. The cause of death was right side hemothorax due to the azygos vein injury by the guide wire. Ai data: present, autopsy data: present. For the purpose of hemodialysis, a long-term dialysis catheter was inserted into the right internal jugular vein with the assistance of landmark method. The guide wire was inserted as far as 30 cm, with no feeling of resistance. After the intervention, wheezing occurred. On the chest X-ray for the confirmation of the position of the catheter, cardiomegaly and right pleural effusion were observed, which was diagnosed as an exacerbation of heart failure. An urgent hemodialysis was performed for the purpose of removing the tissue water. Soon after the start of dialysis, with abnormal intense body motion, cardiopulmonary arrest occurred, resulting in death.
Case 7	The patient had suffered from myelodysplastic syndrome. And was also under the treatment of maintenance hemodialysis for chronic renal failure and tube feeding (gavage) for quadriplegia. Difficulty in communication. The cause of death was suggested as mediastinal hematoma and hemothorax due to the vascular injury. Ai data: absent, autopsy data: absent. For the purpose of replacing the long-term dialysis catheter, the intervention to the left internal jugular vein was performed with the aid of X-ray fluoroscopy and of the real-time ultrasound-guided method. The carotid artery was punctured. After the temporary hemostasis obtained, the intervention to the same left internal jugular vein was performed again. At that time, resistance was felt at the insertion of the guide wire, so its position was confirmed by X-ray fluoroscopy, then a catheter was inserted 30 cm. Hemorrhage continued from the insertion site, but on the next day hemostasis was confirmed. Respiratory condition changed during dialysis on the second day after the insertion, and mediastinal hematoma was confirmed on the third day by CT. The patient died on the seventh day after the intervention.
Case 8	The patient had been under the treatment of maintenance hemodialysis for chronic renal failure. V-P shunt was inserted for subarachnoid hemorrhage. The cause of death was suspected of mediastinal hematoma due to extravascular catheter placement. Ai data: present, autopsy data: absent. For the hemodialysis, a long-term dialysis catheter was inserted to the left internal jugular vein with X-ray fluoroscopy and real-time ultrasound-guided method. Although the reverse blood flow through the catheter was not observed, the procedure itself was smooth, so it was judged that the catheter was inserted into the target blood vessel. On the next day, hemodialysis was started, and a catheter was used as a blood return route. With the dialysis blood flow rate increased, rolling of the eyes and loss of consciousness appeared, and respiratory arrest followed. Mediastinal hematoma was observed on chest X-ray, and the patient died one hour later.
Case 9	The patient had to discontinue the tube-feeding, due to an acute exacerbation of interstitial pneumonia and gastroduodenal ulcer. Difficulty in communication. The definite cause of death is unknown. Regarding the insertion of CVC, the catheter tip placed in the retroperitoneum was suspected as a factor of exacerbation of the general condition. Ai data: absent, autopsy data: absent. For the purpose of intravenous hyper-alimentation, the insertion of CVC (double lumen) from the right inguinal region was done with the assistance of landmark method, but the insertion was difficult due to the collapse of the vein. After multiple punctures the catheter was inserted from the left inguinal region and was checked with the abdominal X-ray. Twelve hours after the start of drip infusion, the patient fell into shock. A distention and mild pain in lower abdomen were observed. Abdominal CT revealed that the catheter tip was inserted into the retroperitoneum and drip infusion was discontinued. An examination puncture to abdominal space was performed, and there was no sign of perforative peritonitis. Conservative treatment was continued, but the condition gradually worsened, and the patient died four days after the insertion.
Case 10	The patient had suffered from advanced cancer and ileus, taking antiplatelet drugs for arteriosclerosis obliterans. The cause of death was suspected as a cerebral haemorrhagic infarction due to the hematogenous metastasis of cancer. Ai data: absent, autopsy data: absent. For the purpose of intravenous hyper-alimentation, CVC was done to the right internal jugular vein with the aid of real-time ultrasound-guided method. After the confirmation with chest X-ray, drip infusion was started using an infusion pump. Approximately 9 hours after the insertion, the patient complained of difficulty in breathing, subsequently cough and chest pain occurred, and pneumothorax was pointed out. Therefore, a chest drainage tube was inserted. Two days after the insertion, pulsatile backflow of blood was observed during the exchange of the drip infusion line. CT revealed that the catheter had penetrated the internal jugular vein and the subclavian artery and dwelled in the aorta. The catheter was removed successfully without major bleeding, which was done with platelet transfusion and under the cardiovascular surgeon on standby. Approximately one month later, the patient died of complications related to the original disease.

**Table 3:** Baseline data for closed-claim cases and reported cases*.

		CCs, n = 37		RCs, n = 10
		Non-death cases, n = 25	Death cases, n = 12	All death cases
Patient				
Age (yr)	X ≦ 19	2	0	0
	20 ≦ X ≦ 39	4	0	0
	40 ≦ X ≦ 59	9	4	1
	60 ≦ X	10	8	8
Sex	Male	12	6	4
	Female	13	6	6
Disease for CVC^†^	Cancer	7	6	1
	Bowel disease	4	2	2
	Renal failure	2	1	3
	Others	12	3	4
Reason for CVC^‡^	Nutrition	13	11	4
	Hemodialysis	1	0	3
	Others	11	1	3
Insertion site^§^	Jugular	4	5	7
	Subclavian	15	5	2
	Femoral	4	2	1
Hospital				
Hospital type	Advanced	2	3	—
	General	22	9	—
	Others	1	0	—
Discipline	Internal medicine	10	5	—
	Surgery	9	6	—
	Anesthesiologist	5	0	—
	Others	1	1	—
Experience (years) ^||^	0 ≦ X < 2	2	2	—
	2 ≦ X < 5	7	0	—
	5 ≦ X < 10	3	1	—
	10 ≦ X	6	4	—

*Only the disease indicated for CVC, the reason for CVC, and the insertion site were published in the reported cases. The age of one case in the reported cases was not disclosed. Aggregated data for sex were disclosed.

^†^No significant differences were observed in the disease indicated for CVC (p = 0.26).

^‡^Significant differences were observed in CVC (p = 0.03). In the RCs, for one patient who died of pneumothorax, it was difficult to secure blood access, and the catheter was inserted into either the jugular vein or subclavian vein. The site of puncture that caused the occurrence of pneumothorax could not be determined from the report. However, it was eventually classified as subclavian because the risk factor for pneumothorax due to internal jugular vein puncture, such as emaciation, was not described in this case.

^§^Two of the non-death cases in the CCs were excluded from the analysis due to the lack of data on the anatomic insertion site. No significant differences were observed in terms of the insertion site (p = 0.28).

^||^ Seven of the non-death cases and five of the death cases in CCs were excluded from the analysis due to the lack of accurate descriptions of experiences after obtaining a physician license.

CC, closed-claim case; CVC, central venous catheterization; RC, reported case.

Of the 37 CCs, 12 were death cases (32.4%), whereas all 10 RCs were death cases. Of the 12 death cases in the CCs, cancer was the most common disease indicated for CVC in 6 cases (50%), and nutrition was the most common reason for insertion in 11 cases (91.6%). Of the 10 RCs, renal failure was the most common disease indicated for CVC in 3 cases (30.0%), and nutrition was the most common reason for insertion in 4 cases (40.0%). Comparison of CCs and RCs demonstrated no significant difference in the disease indicated for CVC (p = 0.26); however, a significant difference was observed in the reason for CVC (p = 0.03). Among the anatomic sites for CVC, the internal jugular vein was the most common insertion site in 5 CCs (41.7%) and 7 RCs (77.8%), with no significant differences between the CCs and RCs (p = 0.28). In the non-death CCs, the subclavian vein was the most common anatomic site, which was reported in 15 cases (65.2%). In the CCs, the catheter was inserted based on the landmark in all 37 cases, regardless of the anatomic site. In the RCs, the catheter was inserted under ultrasound guidance in 6 of the 10 cases, and the anatomic site was the internal jugular vein in all 6 cases. In the RCs in which the internal jugular vein was selected as the CVC site, catheterization was performed based on the landmark in only 1 case. In non-death CCs, 9 cases (50.0%) involved physicians with <5 years of experience after obtaining a physician license versus their involvement in 2 death CCs (28.6%). In addition, restriction of the physicians with <2 years of experience revealed 2 non-death CCs (11.1%) and 2 death CCs (28.6%). None of the CCs involved an anesthesiologist.

### Vascular access and use/maintenance related to CVC according to error type

**Fig 1:**
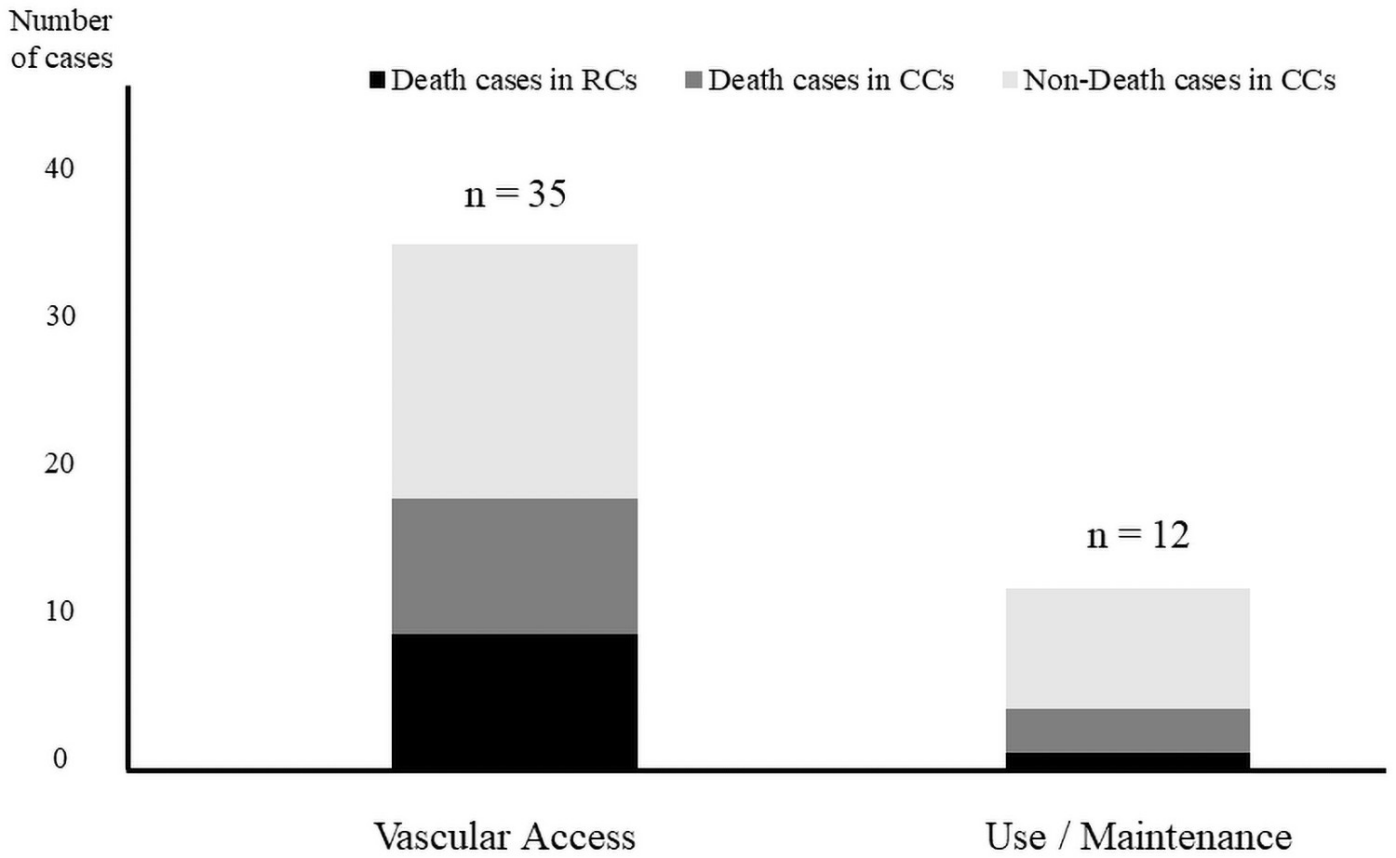
Number of CCs or RCs related to vascular access or to use/maintenance classified according to error type. In CCs, no significant difference was observed between the proportion of deaths due to errors in obtaining vascular access and that due to use/maintenance (p = 1.00). CC, closed-claim case; RC, reported case.

The error type of CVC-related accidents included vascular access in 35 cases (74.5%) and use/maintenance in 12 cases (25.5%) in CCs and RCs (Fig 1).

Of the 35 cases related to vascular access, 18 were deaths (51.4%), including 9 CCs and 9 RCs. Of the 12 cases related to use/maintenance, 4 were deaths (33.3%), including 3 CCs and 1 RC. Among the CVC-related accident cases, most were related to vascular access than to use/maintenance, and the ratio of death cases was also higher.

### Relationship between the anatomic insertion site and complication type in death cases

The anatomic insertion site and fatal complication types are presented in Table 4.

**Table 4:** Complication types related to vascular access in the death cases*.

		Complication type					Total
		Pneumothorax	Hemopneumothorax	Vascular injury	Hematoma	Others	
CCs, n = 9	Jugular	0	1	3(3)	1	0	5
	Femoral	0	0	0	0	1	1
	Subclavian	3	0	0	0	0	3
RCs, n = 9	Jugular	0	1(1)	3(2)	3(3)	0	7
	Femoral	0	0	0	0	1	1
	Subclavian	1^†^	0	0	0	0	1

* Numbers in parentheses indicate the number of patients with coagulopathy.

^†^In the RCs, for the patient who died of pneumothorax, blood access was difficult to secure, and insertion was made into either the jugular vein or the subclavian vein. The site of puncture responsible for the occurrence of pneumothorax could not be determined from the report; however, it was eventually classified as subclavian because the risk factor for pneumothorax due to internal jugular vein puncture, such as emaciation, was not described in this case.

CC, closed-claim case; RC, reported case.

The internal jugular vein was the most common anatomic insertion site that resulted in fatal complications, with 5 (55.6%) of 9 cases in CCs and 7 (77.7%) of 9 cases in RCs. No differences were observed between the fatal complication types in CCs versus RCs. In the death cases related to internal jugular vein insertion, the complications of vascular injury occurred in 3 of 5 CCs (60.0%) and 3 of 7 RCs (42.9%). In the death cases due to internal jugular vein insertion, coagulopathy was observed in 3 of 5 CCs (60.0%) and 6 of 7 RCs (85.7%). In the vascular injury cases, coagulopathy was observed in all CC cases and 2 of 3 RCs (66.7%).

## Discussion

This study aimed to compare and analyze CVC-related CCs and CVC-related RCs to identify points in the Medsafe Japan and ISC recommendations that should be emphasized specifically to prevent the recurrence of similar CVC-related death. In comparing the deaths of CCs and RCs, incidents in vascular access in jugular catheterization were observed as common characteristics. In the analysis of complication types, fatal bleeding complications caused by jugular catheterization were also observed as a common characteristic. In the death cases due to internal jugular vein puncture, coagulopathy was observed in 9 of the 12 death cases (75.0%). In the analyses of CCs, there were no death cases in which anesthesiologist performed CVC. When the practice of CVC to the internal jugular vein was compared between the death CCs and RCs, the ultrasound-guided method was used in 6 of 7 RCs (85.7%), whereas the landmark method was used in all 37 CCs.

Ultrasound guidance is safer than the landmark technique because physicians use ultrasound to guide the catheter placement [5, 25–27]. In recent years, the internal jugular vein has often been recommended as the first-line option in the selection of an anatomic site for catheter insertion. One of the reasons for this selection is that it is easier to access the jugular vein during CVC because the ultrasound-guided method enables direct observation. In addition, jugular catheterization can be performed to minimize the risk of pneumothorax compared with subclavian catheterization. It can also avoid infectious diseases more than femoral catheterization [6–8, 26]. Therefore, recommendations published by Medsafe Japan and ISC describe in detail the appropriate puncture technique under ultrasound guidance at the jugular vein, which can be expected to reduce CVC-related deaths by incorporating this into clinical practice. Some deaths in CCs may have been avoided using the ultrasound-guided method. However, bleeding complications in CVC cannot be completely avoided, even if catheterization is performed with appropriate use of the ultrasound-guided method. In the death cases due to internal jugular vein puncture, concomitant coagulopathy is conspicuous, and fatal complications due to bleeding may occur regardless of the use of ultrasound guidance. The risk of bleeding complications at CVC in patients with coagulopathy is well recognized among physicians. However, data indicate that vascular injury that occurs in patients with coagulopathy due to internal jugular vein puncture results in fatal complications. In unforeseen death cases, physicians may have been overconfident in ultrasound guidance and may not have seen the risk of bleeding seriously. Therefore, concomitant coagulopathy seems to be a critical risk of CVC-related death. CVC with appropriate ultrasound-guided method is important in avoiding CVC-related death but may be insufficient.

In Japan, anesthesiologists are mainly responsible for CVC in some specialized hospitals, whereas in many general hospitals, general physicians, including surgeons, are responsible for CVC [28]. Given that the guidelines for CVC have been published by anesthesiology societies in various countries, that anesthesiologists are highly experienced in performing CVC, and that none of the death CCs involved anesthesiologists in this study, anesthesiologists generally seem to have a deep knowledge of the safe performance of a CVC, including ensuring a safe puncture environment. Therefore, CCs and RCs may present a risk that is likely to be overlooked by non-anesthesiologists responsible for CVC. The results of this study suggest that physicians, especially non-anesthesiologists, may overlook the fatal bleeding risk, although they may have an abstract understanding that patients with coagulopathy have a risk of bleeding that can lead to death. Therefore, all physicians involved in CVC should accurately observe the risk of internal jugular insertion in patients with coagulopathy. The risks of CVC performed in the internal jugular vein in patients with coagulopathy should be reconsidered.

To establish the Medsafe Japan and ISC recommendations more effectively, this study emphasizes the following points. First, the indications for CVC in the internal jugular vein for patients with coagulopathy should be carefully reconsidered. In this context, item 1 of the recommendations that addresses the risk of a patient with coagulopathy should be greatly emphasized. Second, because vascular injury, hemopneumothorax, and hematoma can occur as unforeseen fatal complications, CVC in the internal jugular vein for patients with coagulopathy under ultrasound guidance should be performed in environments capable of addressing such complications. If this secure environment cannot be established, then CVC in the internal jugular vein for patients with coagulopathy should be temporarily postponed.

This study has several limitations. First, the recommendations were first published soon after the initiation of the Medical Accident Investigation System. This system is continuously evolving and accumulating more cases, and many more cases are currently available. Therefore, new findings may be obtained in the reanalysis of CVC-related RCs. Second, this study was a retrospective review of CCs provided by one department of SJ and did not represent all CVC-related medical claims. Therefore, the results may only be applicable to a single aspect of malpractice claims. Despite these limitations, this study was the first to evaluate the Medsafe Japan and ISC recommendations based on unforeseen deaths related to CVC in the Medical Accident Investigation System in Japan. The clinical management of diseases is expected to increase based on specialization and complexity, and it will be necessary to ensure patient safety in the future. We hope that the findings of the present study will help physicians responsible for CVC to better understand the need for preventing CVC-related fatal complications.
